# Secondary Metabolites from Australian Lichens *Ramalina celastri* and *Stereocaulon ramulosum* Affect Growth and Metabolism of Photobiont *Asterochloris erici* through Allelopathy

**DOI:** 10.3390/molecules29194620

**Published:** 2024-09-29

**Authors:** Martin Bačkor, Dajana Kecsey, Blažena Drábová, Dana Urminská, Martina Šemeláková, Michal Goga

**Affiliations:** 1 Department of Biochemistry and Biotechnology, Institute of Biotechnology, Faculty of Biotechnology and Food Sciences, Slovak University of Agriculture, Tr. A. Hlinku 2, 949 76 Nitra, Slovakia; blazena.drabova@uniag.sk (B.D.); dana.urminska@uniag.sk (D.U.); 2 Department of Botany, Institute of Biology and Ecology, Faculty of Science, Pavol Jozef Šafárik University in Košice, Mánesova 23, 041 67 Košice, Slovakia; dajana.rucova@upjs.sk (D.K.); michal.goga@upjs.sk (M.G.); 3 Department of Medical Biology, Faculty of Medicine, Pavol Jozef Šafárik University in Košice, Trieda SNP 1, 040 11 Košice, Slovakia; martina.semelakova@upjs.sk

**Keywords:** allelopathy, algae, antioxidants, citric acid, glutathione, lichens, Krebs cycle, organic acids, phenolic compounds, secondary metabolites

## Abstract

In the present work, the phytotoxic effects of secondary metabolites extracted from lichen *Ramalina celastri* (usnic acid) and lichen *Stereocaulon ramulosum* (a naturally occurring mixture of atranorin and perlatolic acid, approx. 3:1) on cultures of the aposymbiotically grown lichen photobiont *Asterochloris erici* were evaluated. Algae were cultivated on the surface of glass microfiber disks with applied crystals of lichen extracts for 14 days. The toxicity of each extract was tested at the two selected doses in quantities of 0.01 mg/disk and 0.1 mg/disk. Cytotoxicity of lichen extracts was assessed using selected physiological parameters, such as growth (biomass production) of photobiont cultures, content of soluble proteins, chlorophyll *a* fluorescence, chlorophyll *a* integrity, contents of chlorophylls and total carotenoids, hydrogen peroxide, superoxide anion, TBARS, ascorbic acid (AsA), reduced (GSH) and oxidized (GSSG) glutathione, and composition of selected organic acids of the Krebs cycle. The application of both tested metabolic extracts decreased the growth of photobiont cells in a dose-dependent manner; however, a mixture of atranorin and perlatolic acid was more effective when compared to usnic acid at the same dose tested. A higher degree of cytotoxicity of extracts from lichen *S. ramulosum* when compared to identical doses of extracts from lichen *R. celastri* was also confirmed by a more pronounced decrease in chlorophyll *a* fluorescence and chlorophyll *a* integrity, decreased content of chlorophylls and total carotenoids, increased production of hydrogen peroxide and superoxide anion, peroxidation of membrane lipids (assessed as TBARS), and a strong decrease in non-enzymatic antioxidants such as AsA, GSH, and GSSG. The cytotoxicity of lichen compounds was confirmed by a strong alteration in the composition of selected organic acids included in the Krebs cycle. The increased ratio between pyruvic acid and citric acid was a very sensitive parameter of phytotoxicity of lichen secondary metabolites to the algal partner of symbiosis. Secondary metabolites of lichens are potent allelochemicals and play significant roles in maintaining the balance between mycobionts and photobionts, forming lichen thallus.

## 1. Introduction

Lichens are self-sustaining ecosystems forming a vegetative body (thallus) composed of a taxonomically defined fungal partner (mycobiont) and an extracellular arrangement of minimally one photosynthetic partner (photobiont), which is typically represented by algae or/and cyanobacteria. Within the “lichen” ecosystem, an indeterminate number of other microscopic organisms is typically present, representing the remaining components of the thalli, e.g., cystobasidiomycete yeast. However, it seems that the presence of cystobasidiomycete yeasts in lichen thalli is much less mycobiont-specific than the photobionts [1]. Lichens are, for this reason, considered the symbiotic phenotypes of lichen-forming fungi [2], and lichen names are determined by the presence of the fungal partner. The resulting “organism” does not resemble parental organisms when cultivated as a single symbiont in the laboratory [3], and the same mycobiont may produce a different phenotype when associated with a different photobiont [4].

Lichen plays an important role in the nutrient cycle. They can also be an important part of food chains as many herbivores feed on them, including gastropods, nematodes, mites, or even some mammalian herbivores, e.g., reindeer. They can grow on diverse surfaces and occur from sea level to high alpine elevation, from the polar regions to the equator. They have an important function in boreal forest ecology as they can affect the metabolism of plants that form biocenoses with them [3]. Lichens grow in diverse environments and, due to their symbiotic nature, can survive in extreme environments as extremophiles. Certain species can grow and develop in habitats affected by excessive cold, heat, drought, UV radiation, or metal pollution, where individual organisms forming lichen thallus can only barely survive [3].

A very important aspect of lichen-forming symbiosis is the production of more than one thousand substances that are unique to this group of organisms and are called secondary metabolites [5]. They usually constitute from 0.1 to 5.0% (*w*/*w*) of thallus dry weight and include dibenzofuran derivatives, depsides, and depsidones. Shortly, these metabolites are predominantly of mycobiont origin, extracellular phenols localized on the surfaces of mycobiont hyphae, and have many biological and ecological functions, e.g., antiviral and antimicrobial activities, allelopathy, antiherbivory, UV and excessive light screening of photobionts, and chelating of heavy metals [5,6].

Most of the lichen secondary metabolites, including types of depsides, depsidones, depsones, and dibenzofurans, are derived from the polyketide pathway, previously known as the acetyl-polymalonyl pathway [7]. Usnic acid (UA) is a yellowish pigment and dibenzofuran derivative, one of the most common cortical secondary metabolites of lichens, especially abundant in members of the genera *Alectoria*, *Usnea*, *Cladonia,* and *Xanthoparmelia*. Atranorin (ATR) is a depside and another widespread cortical secondary metabolite abundant in members of the lichen genera *Parmotrema*, *Cladonia,* or *Stereocaulon*. Perlatolic acid (PA) also belongs to lichen depsides; however, when compared to UA and ATR, localized in the medullar part of lichen thalli, PA is typically present in lichens in considerably lower quantities when compared to cortical compounds and present, for instance, in the lichen genera *Cladonia* and *Stereocaulon* [8,9].

So far, UA, ATR, and PA have previously been tested for their antibiotic, antiviral, antimycotic, antiprotozoal, antiherbivore, antiproliferative, anti-inflammatory, analgesic, antipyretic, allelopathic, and UV-protecting effects [5,10]. UA and ATR play significant photoprotective roles for symbiotic photobionts in lichen thalli; however, compound-specific screening mechanisms are still not sufficiently understood [11,12].

The mechanisms of the allelopathic effects of lichen secondary metabolites on plants in biocenoses containing lichens are still not sufficiently researched. Since the minimum requirement for a functional lichen thallus is the presence of mycobiont hyphae, which frequently produce typical lichen phenolic substances and photobiont cells, the involvement of lichen secondary metabolites in potential regulation of photobiont biology and ecology through allelopathy is another fascinating task for modern experimental plant biology. 

As lichen photobionts are in direct contact with cortical and medullar extracellular metabolites produced by mycobiont, the main aim of this study was to evaluate their potential allelopathic effects on aposymbiotically grown lichen photobiont cultures of *A. erici* during 2-week prolonged cultivation on the surface of glass fiber disks coated with crystals of lichen secondary metabolites. Secondary metabolites extracted from lichen *Ramalina celastri* (usnic acid) and lichen *Stereocaulon ramulosum* (the naturally occurring mixture of atranorin and perlatolic acid, approx. 3:1) were used at two selected doses, in quantities of 0.01 mg/disk and 0.1 mg/disk. Allelopathic effects of lichen extracts were assessed using selected physiological parameters, including growth (biomass production) of photobiont cultures, content of soluble proteins, chlorophyll *a* fluorescence, chlorophyll *a* integrity, contents of chlorophylls and total carotenoids, hydrogen peroxide, superoxide anion, TBARS production, content of ascorbic acid (AsA), reduced (GSH), and oxidized (GSSG) glutathione, or composition of selected organic acids included in the Krebs cycle.

## 2. Results and Discussion

### 2.1. Photobiont Growth

**Figure 1:**
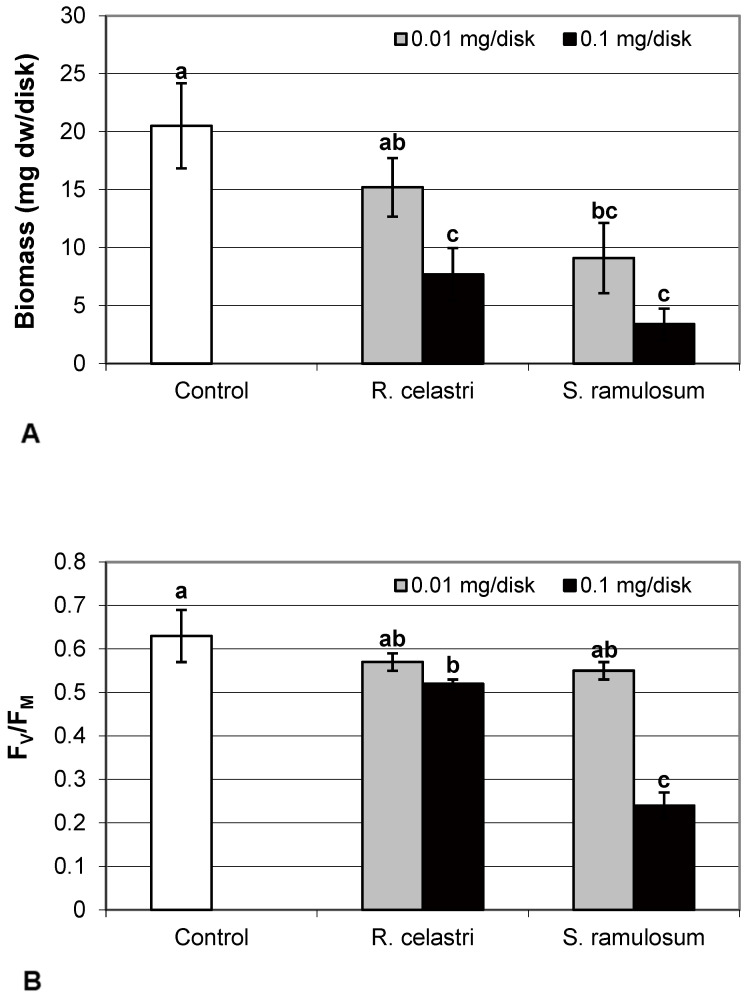

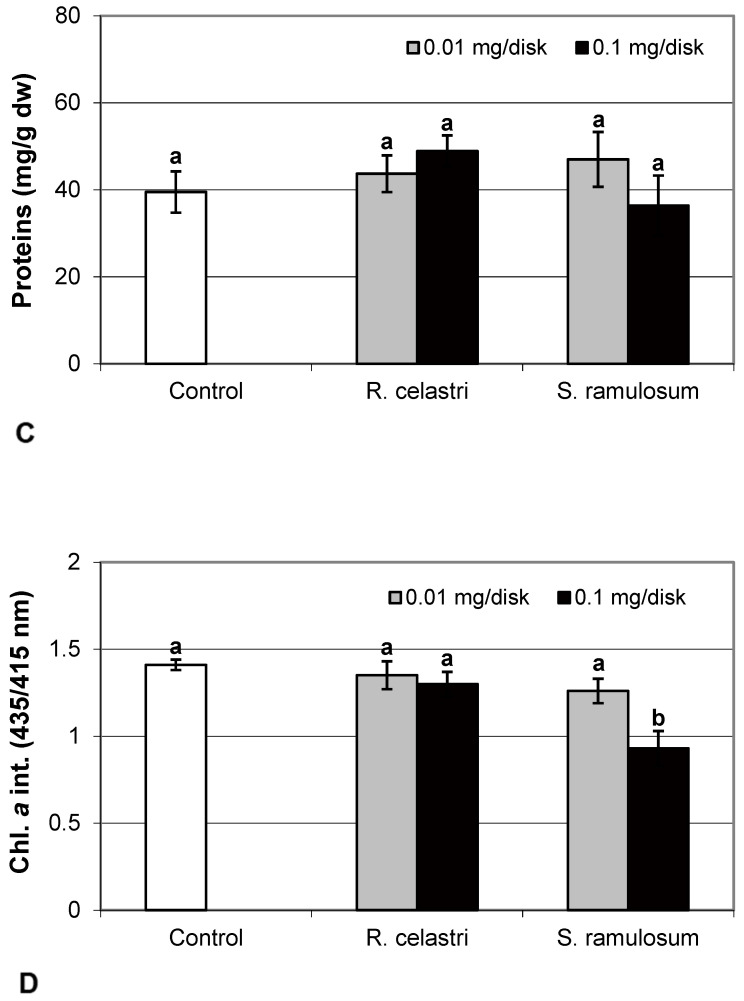
Biomass production ((**A**); mg dw/disk), chlorophyll *a* fluorescence ((**B**); F_V_/F_M_), content of soluble proteins ((**C**); mg/g dw), and chlorophyll *a* integrity ((**D**); A 435 nm/415 nm) of 2 week-old photobiont *Asterochloris erici* cultures cultivated on disks with addition of secondary metabolites extracts from lichens *Ramalina celastri* and *Stereocaulon ramulosum* (0.01 and 0.1 mg/disk). Values in vertical columns followed by the same letter(s) are not significantly different according to Tukey’s test (*p* < 0.05), *n* = 3.

Biomass production (mg dw/disk) of two-week-old *A. erici* cultures decreased by the presence of extracts from *R. celastri* and. *S. ramulosum* in a quantitatively dependent manner (Figure 1A). While both tested quantities of *S. ramulosom* extracts significantly decreased photobiont growth, the lower tested amount of extract from *R. celastri* (0.01 mg/disk) was not strong enough to be significant. 

Secondary metabolites of lichens play many important biological and ecological roles. Mechanisms of their action in cases of antiproliferative or antibiotic effects are now relatively well documented for some common lichen cortical compounds. It has been previously found that both cortical lichen compounds used in the present study are potent inhibitors of the growth and development of some cancer cell lines. The suppression of viability and cell proliferation caused by the application of UA and ATR correlated strongly with an increased number of floating cells and a higher apoptotic index. The analysis of cell cycle distribution also revealed an accumulation of cells in S-phase [13].

Mechanisms of allelopathic role of lichen secondary metabolites are, on the other hand, still far from their full understanding. Allelopathy in lichens is probably a very complex phenomenon, and it appears that secondary metabolites of lichens from different chemical groups differ in the degree of their phytotoxicity. Firstly, lichen secondary metabolites on a global scale may influence boreal forest ecology through allelopathy [14] and can directly affect plant metabolism, e.g., confirmed in the case of seedlings of Norway spruce and Scots pine seedlings, or indirectly through allelopathic effects on soil mycorrhizal fungi [14]. UA also has phytotoxic acid on cultures of lower plants, e.g., mosses, which typically form phytocenoses with lichens [15]. Secondly, the lichen thallus itself is formed as an ecosystem, and in addition to its own photobiont cells growing within its thallus, many other microscopic organisms, including cystobasidiomycete yeasts, may also be present [2]. It has been found in our previous studies that some lichen metabolites can directly decrease the growth of algal cultures, including lichen photobionts [16,17]. It can be the result of the effect of some lichen compounds (e.g., UA) on the spindle apparatus during the processes of mitosis [18,19]. However, the efficiency of lichen compounds in control of photobiont cell division is different, and some cortical metabolites can positively affect photobiont cells through their photoprotective roles, screening UV radiation, and excessive light-causing photoinhibition in photobiont cells [11,12].

### 2.2. Activity of Photosystem II, Soluble Proteins, and Content of Assimilation Pigments in Photobiont

Chlorophyll *a* fluorescence (expressed as F_V_/F_M_ ratio) of two-week-old *A. erici* cultures decreased by the presence of the highest tested (0.1 mg/disk) amount of extracts from *R. celastri* and *S. ramulosum* (Figure 1B). The inhibition effect was more pronounced after exposure of algal cells to *S. ramulosum* extracts. The content of the soluble proteins (mg/g dw) in *A. erici* was a stable parameter and did not change regardless of the lichen source of tested extracts and extract quantities (Figure 1C).

**Figure 2:**
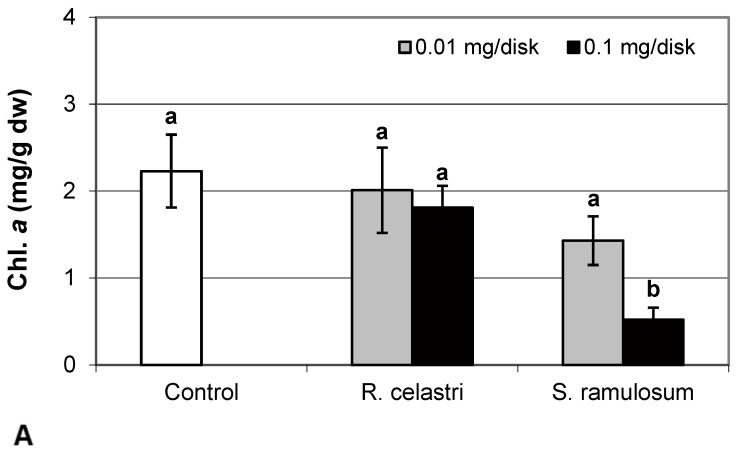

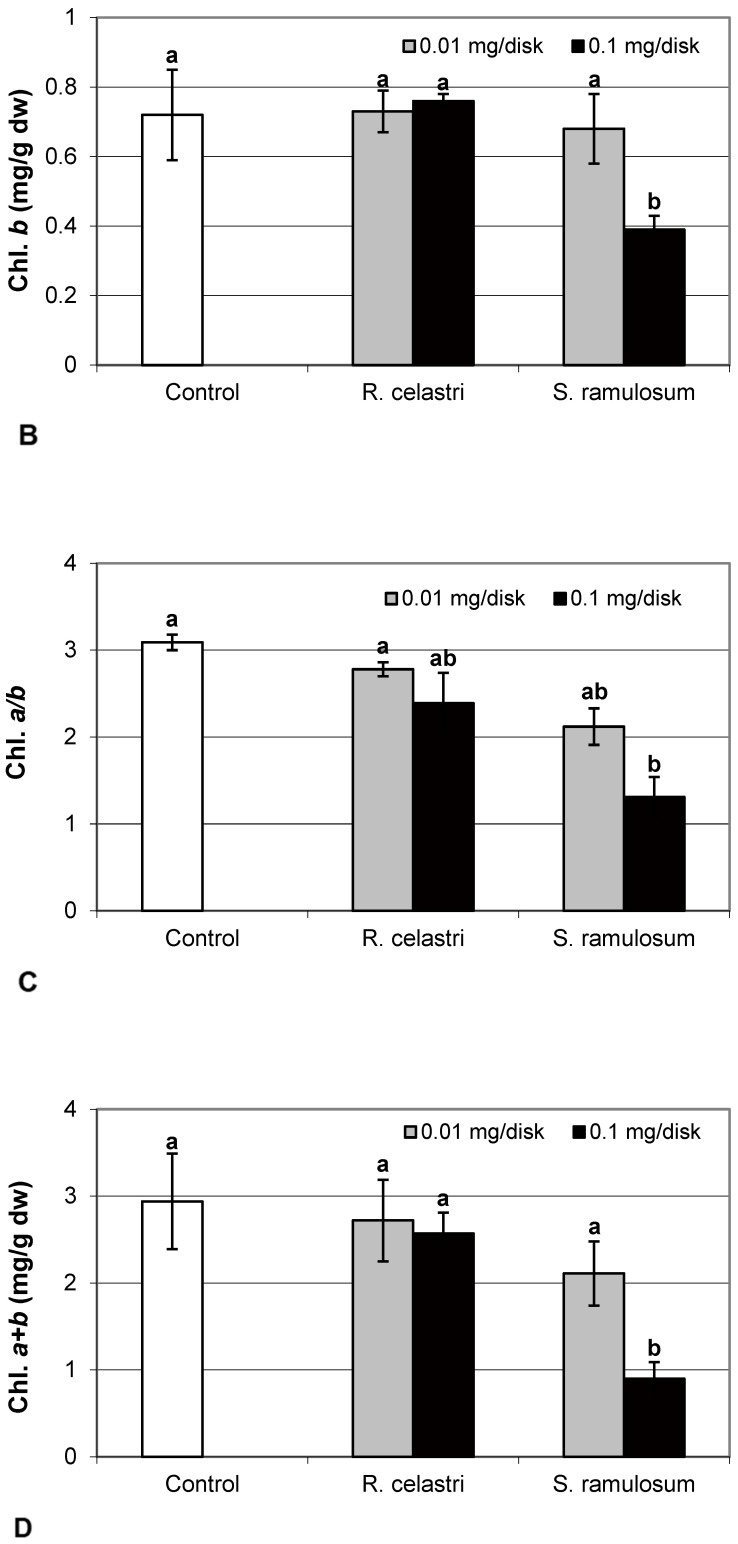
Chlorophyll *a* content ((**A**); mg/g dw), chlorophyll *b* content ((**B**); mg/g dw), chlorophyll *a*/*b* ((**C**), and chlorophyll *a*+*b* content ((**D**); mg/g dw) of 2-week-old photobiont *Asterochloris erici* cultures cultivated on disks with the addition of secondary metabolites extracts from lichens *Ramalina celastri* and *Stereocaulon ramulosum* (0.01 and 0.1 mg/disk). Values in vertical columns followed by the same letter(s) are not significantly different according to Tukey’s test (*p* < 0.05), *n* = 3.

**Figure 3:**
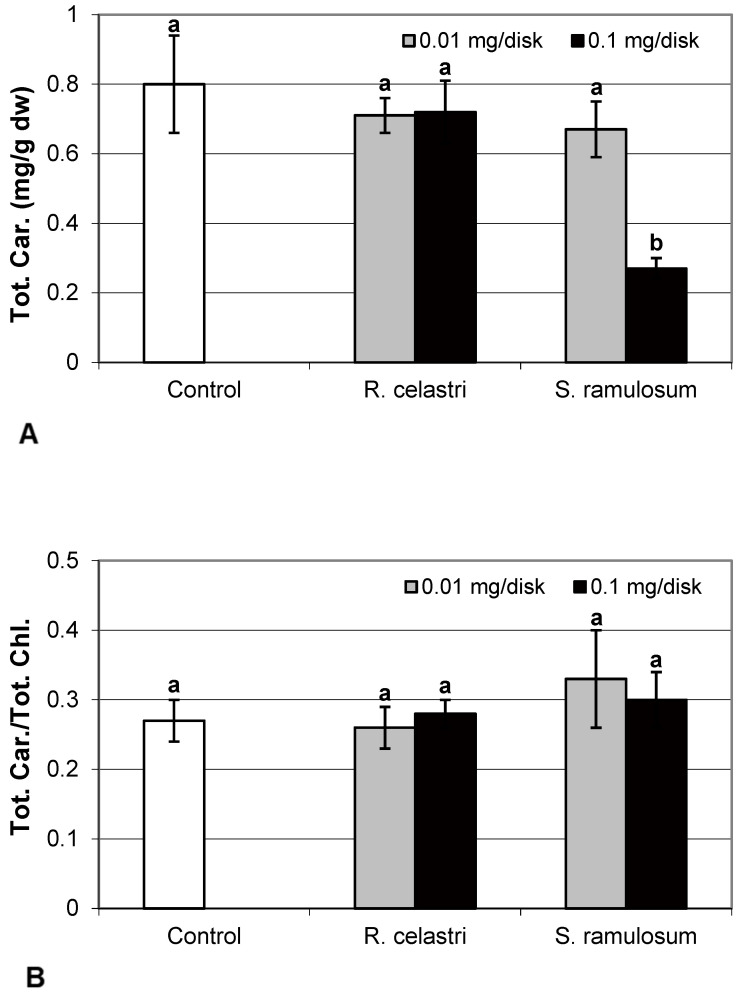
Content of total carotenoids ((**A**); mg/g dw) and total carotenoids/total chlorophyll (**B**) of 2-week-old photobiont *Asterochloris erici* cultures cultivated on disks with the addition of secondary metabolites extracts from lichens *Ramalina celastri* and *Stereocaulon ramulosum* (0.01 and 0.1 mg/disk). Values in vertical columns followed by the same letter(s) are not significantly different according to Tukey’s test (*p* < 0.05), *n* = 3.

Chlorophyll *a* integrity (expressed as a ratio of absorbance of pigment extracts at 435 nm/415 nm) of two-week-old *A. erici* cultures decreased only as a result of the highest tested (0.1 mg/disk) amount of extract from *S. ramulosum* (Figure 1D). Similarly, chlorophyll *a* content (Figure 2A), chlorophyll *b* content (Figure 2B), chlorophyll *a/b* ratio (Figure 2C), chlorophyll *a*+*b* content (Figure 2D), and content of total carotenoids (Figure 3A) of two-week-old *A. erici* cultures decreased only as a result of the highest tested (0.1 mg/disk) amount of extracts from *S. ramulosum*. Contents of chlorophylls and total carotenoids are expressed as mg/g dw of algal biomass. The ratio of total carotenoids/total chlorophyll in *A. erici* was a stable parameter and did not change regardless of the lichen source of tested extracts and extract quantities (Figure 3B).

UA and a mixture of ATR+PA used in this study demonstrated strong inhibition effects on chlorophyll *a* fluorescence. The influence of lichen secondary metabolites on Photosystem II, including UA, was demonstrated previously, and the determination of chlorophyll *a* fluorescence in photobiont cells was found to be a useful marker for the assessment of their physiological status [20,21]. It seems that the utilization of other chlorophyll fluorescence methods may help explain the sensitivity or tolerance of complex photosynthetic processes in lichen algae [22,23]. 

The phytotoxic effect of secondary metabolites can also involve the alteration of assimilation pigment composition. In the previous study, Rapsch and Ascaso [24] studied the effect of medullary lichen secondary metabolite evernic acid on the chloroplast structure of spinach (*Spinacia oleracea*). Decreased total chlorophyll content and chlorophyll *a* content, as well as decreased chlorophyll *a* to chlorophyll *b* ratio due to the presence of evernic acid, was demonstrated also in free-living algae and lichen photobionts [25]. Bačkor et al. [16] found that the assimilation pigments (chlorophyll *a*, chlorophyll *b,* and content of total carotenoids) in the free-living alga *Scenedesmus quadricauda* were strongly decreased due to the presence of UA. However, the sensitivity of photobiont *A. erici* (syn. *Trebouxia erici* in the former study) cells was significantly lower. On the other hand, the assimilation pigments of *A. erici* were sensitive to the presence of a mixture of ATR+PA used in this study. This was also confirmed by decreased chlorophyll *a* integrity, a parameter very frequently employed in studies focused on lichen photobiont stress physiology [3]. 

### 2.3. Oxidative Status and Membrane Lipid Peroxidation in Photobiont

**Figure 4:**
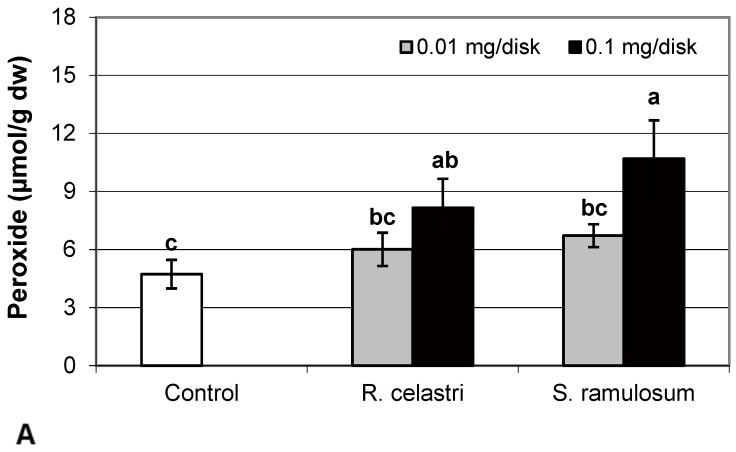

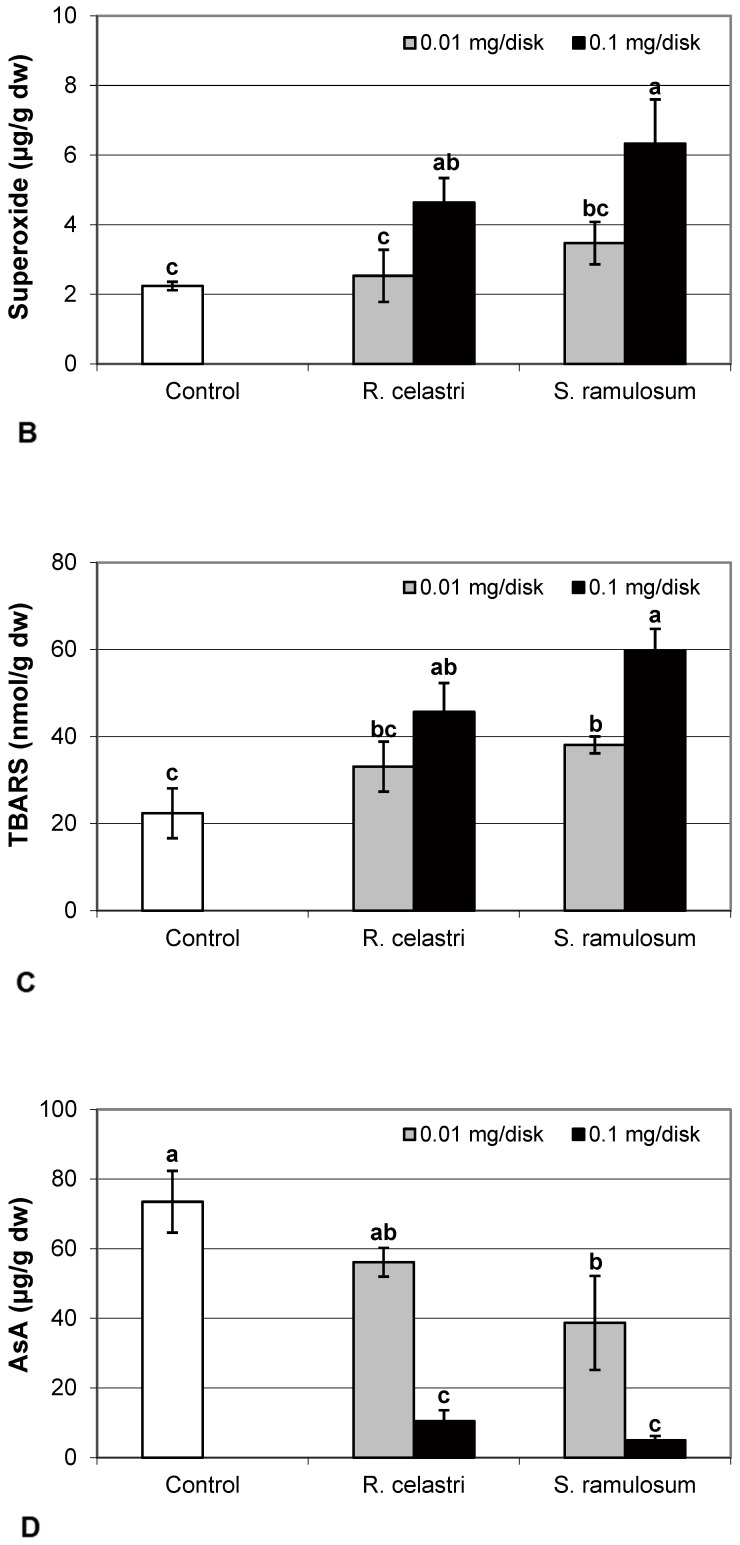
Content of hydrogen peroxide ((**A**); µmol/g dw), superoxide anion ((**B**); µg/g dw), TBARS ((**C**); nmol/g dw), and ascorbic acid (AsA, (**D**); µg/g dw) of 2-week-old photobiont *Asterochloris erici* cultures cultivated on disks with the addition of secondary metabolites extracts from lichens *Ramalina celastri* and *Stereocaulon ramulosum* (0.01 and 0.1 mg/disk). Values in vertical columns followed by the same letter(s) are not significantly different according to Tukey’s test (*p* < 0.05), *n* = 3.

Hydrogen peroxide (µmol/g dw) content (Figure 4A) and content (µg/g dw) of superoxide anion (Figure 4B) of two-week-old *A. erici* cultures increased by the presence of the highest tested (0.1 mg/disk) amount of extracts from *R. celastri* and. *S. ramulosum*. Production of TBARS (nmol/g dw) in two-week-old *A. erici* cultures increased by the presence of extracts from *R. celastri* as well as *S. ramulosum* in a quantitatively dependent manner (Figure 4C). While both tested quantities of *S. ramulosom* extracts significantly increased TBARS production, the increase in TBARS caused by the lower tested amount of extract from *R. celastri* (0.01 mg/disk) was not strong enough to be significant (Figure 4C). 

Oxidative stress may damage many biological molecules, including DNA and cell membrane lipids, which are significant targets of cellular injury (free radical attack). Plant polyphenols are well recognized for their antioxidant activities. Levels of hydrogen peroxide, superoxide, and TBARS significantly increased in photobiont cultures due to the highest tested concentrations of UA and a mixture of ATR + PA. Han et al. [26] found that usnic acid disrupted electron transport in mitochondria and induced oxidative stress in the hepatocytes. Caviglia et al. [27], on the other hand, demonstrated that UA extracted from lichen *Parmelia soredians* may act as an antioxidant and detoxify ROS produced after the application of the herbicide Paraquat. 

Both tested compounds in the present study, UA and ATR, were previously found to be relatively effective anti-cancer compounds [28]. UA and ATR were capable of inducing a massive loss in the mitochondrial membrane potential, along with caspase-3 activation and phosphatidylserine externalization in tested cancer cell lines. The induction of both ROS and RNS was, at least in part, responsible for the cytotoxic effects of these cortical lichen secondary metabolites. Based on the detection of protein expression (PARP, p53, Bcl-2/Bcl-xL, Bax, p38, pp38), it has been found that UA and ATR may be activators of programmed cell death in cancer cell lines, probably through the mitochondrial pathway. 

We know that UA increased levels of hydrogen peroxide and superoxide in the cells of lichen photobiont *Trebouxia erici* [16]. However, studies focused on the production of ROS or degradation of membrane lipids in lichens and their symbionts are still insufficiently intensively examined and require further study. 

### 2.4. Production of Ascorbic Acid (AsA), Reduced (GSH) and Oxidized (GSSG) Glutathione

The content of AsA (µg/g dw) in two-week-old *A. erici* cultures decreased by the presence of a higher tested (0.1 mg/disk) amount of extract from *R. celastri* and both tested amounts of extracts from *S. ramulosum* (Figure 4D). 

**Figure 5:**
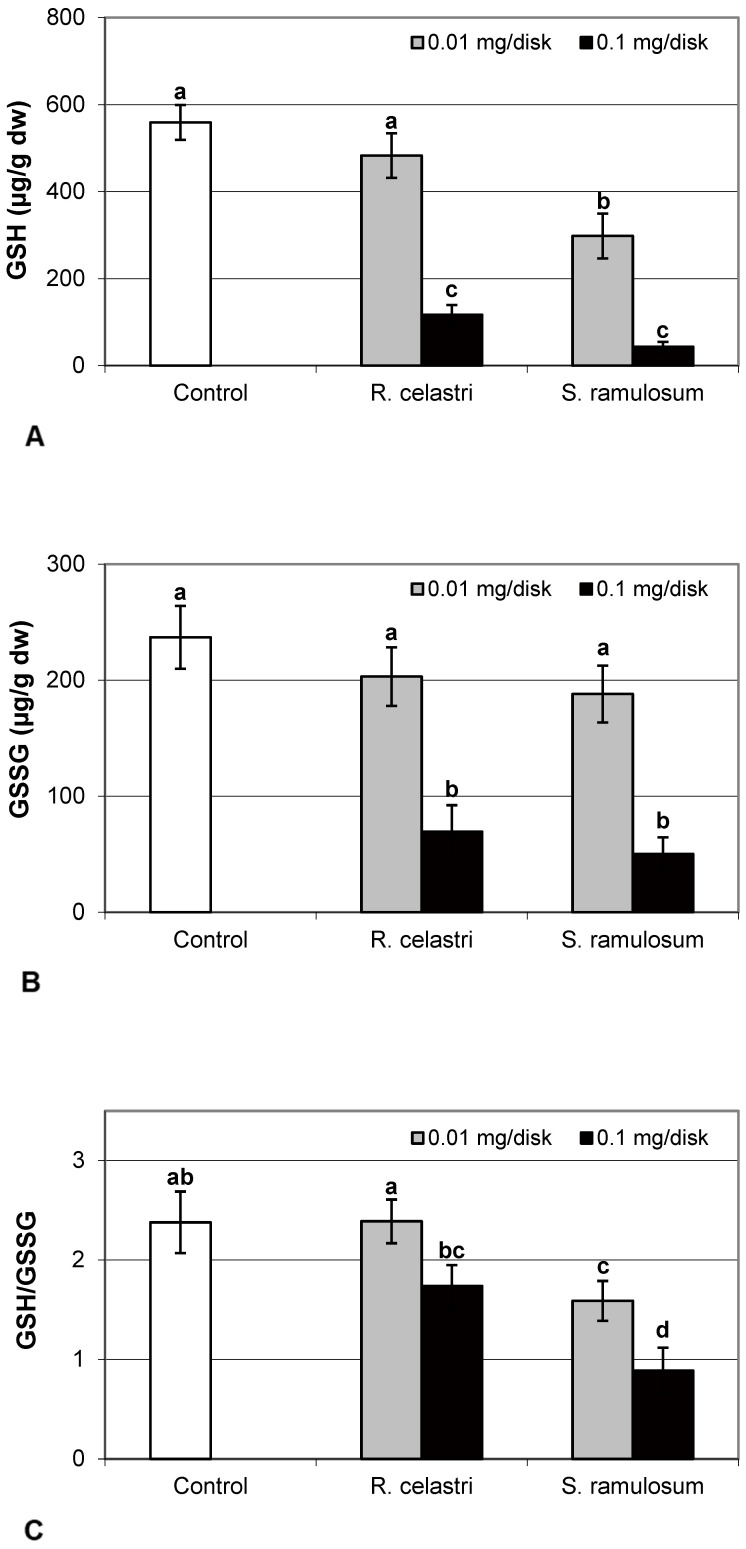

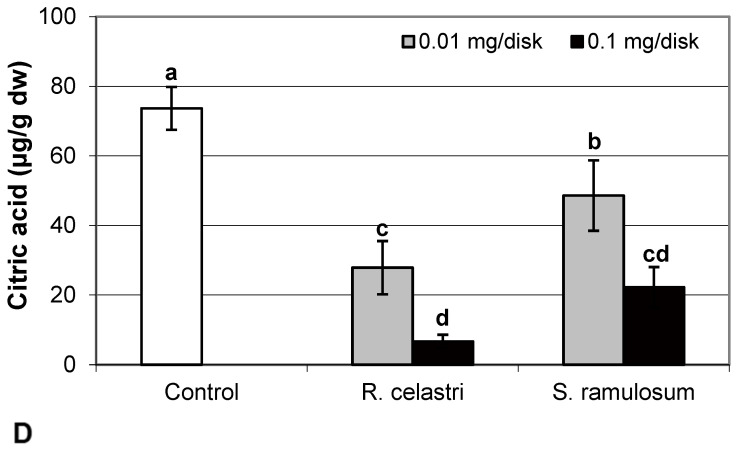
Content of reduced glutathione (GSH, (**A**); µg/g dw), oxidized glutathione (GSSG, (**B**); µg/g dw), GSH/GSSG (**C**), and citric acid ((**D**); µg/g dw) of 2-week-old photobiont *Asterochloris erici* cultures cultivated on disks with the addition of secondary metabolites extracts from lichens *Ramalina celastri* and *Stereocaulon ramulosum* (0.01 and 0.1 mg/disk). Values in vertical columns followed by the same letter(s) are not significantly different according to Tukey’s test (*p* < 0.05), *n* = 3.

The content of GSH and GSSG (µg/g dw) in two-week-old *A. erici* cultures decreased by the presence of lichen extracts on the disk surface (Figure 5A,B). GSH and GSSG content decreased in a dose-dependent manner in both amounts of lichen extracts tested. While a significant decrease in GSH content was observed only after application of the highest tested dose (0.1 mg/disk) of extract from *R. celastri*, both tested amounts of extracts from *S. ramulosum* had a strong inhibition effect on GSH content. GSSG content in algae was inhibited only after higher (0.1 mg/disk) tested doses of extracts from both lichens (Figure 5B). Due to this reason, we observed a significant decrease in the GSH/GSSG ratio in photobiont cells after the application of both tested amounts of extracts from *S. ramulosum* (Figure 5C).

The response of lichen algae to the presence of allelochemicals, which are produced by their symbiotic partners, mycobiont, is still enigmatic. In general, when plants are exposed to biotic and/or abiotic stresses, they increase ROS production, which leads to changes in the balance between ROS and cellular antioxidants [29]. The Glutathione-Ascorbate (GSH-AsA) cycle is stimulated to detoxify ROS and ROS-generated toxic products of plant metabolism. The AsA and GSH are considered an antioxidant buffer to control ROS levels [30]. AsA can also play an important role in the regulation of xanthophyll pigment activity. Glutathione is found in all eukaryotes and is one of the most dominant cellular thiol compounds, which is important in defense against ROS. In the lichen, photobiont cells were found to play a significant role in metal defense [3]. Pools of GSH, GSSG, and AsA in photobiont *A. erici* decreased as a response to secondary metabolites presence on cultivation disks.

### 2.5. Photobiont Organic Acids

In the present study, we also analyzed the content of selected organic acids (µg/g dw) related to the Krebs cycle, or the so-called “citric acid cycle”. Citric acid content (Figure 5D) in two-week-old *A. erici* cultures decreased in a dose-dependent manner in the case of both tested lichen extracts; however, extracts from *R. celastri* were more effective when compared to identical amounts of extracts from *S. ramulosum*.

**Figure 6:**
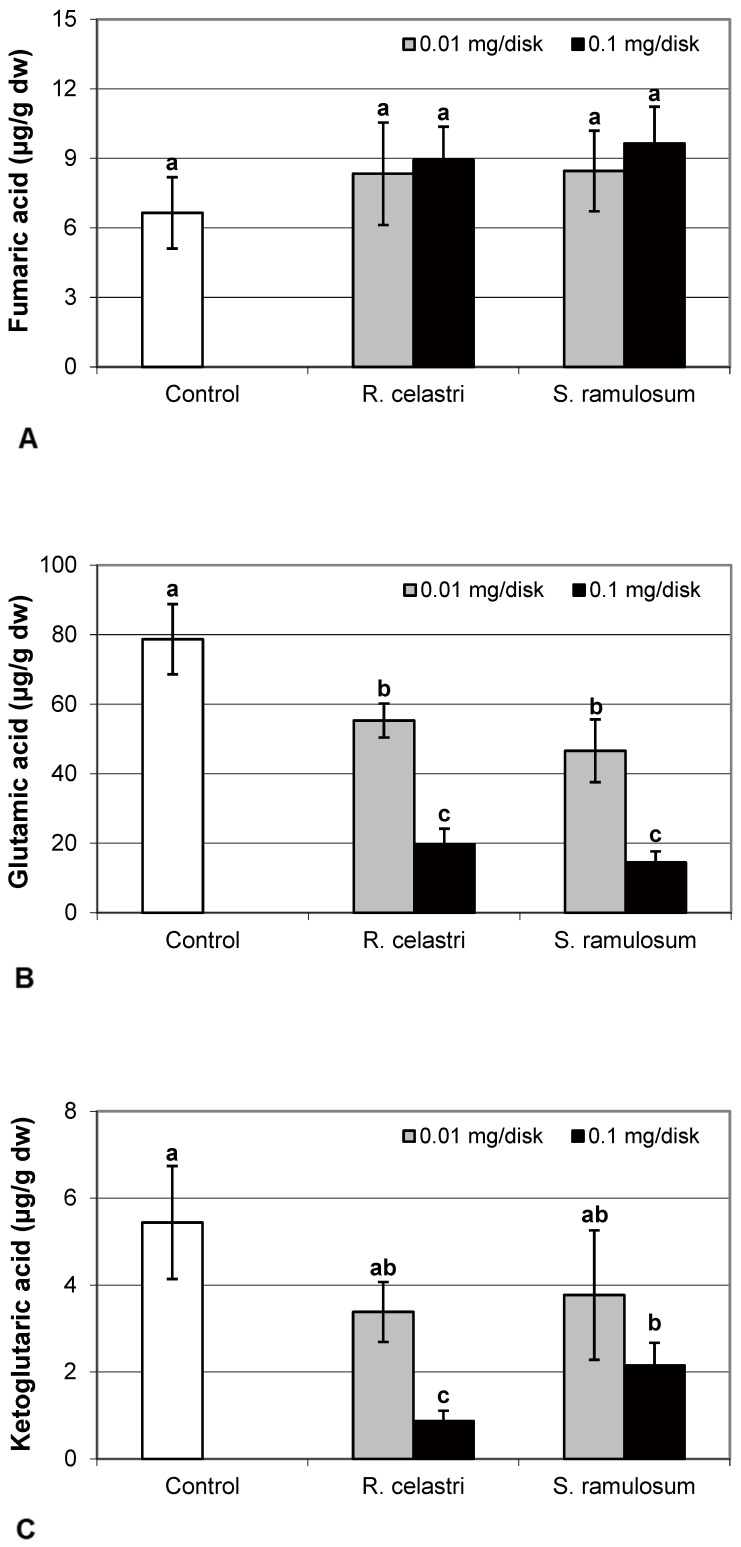

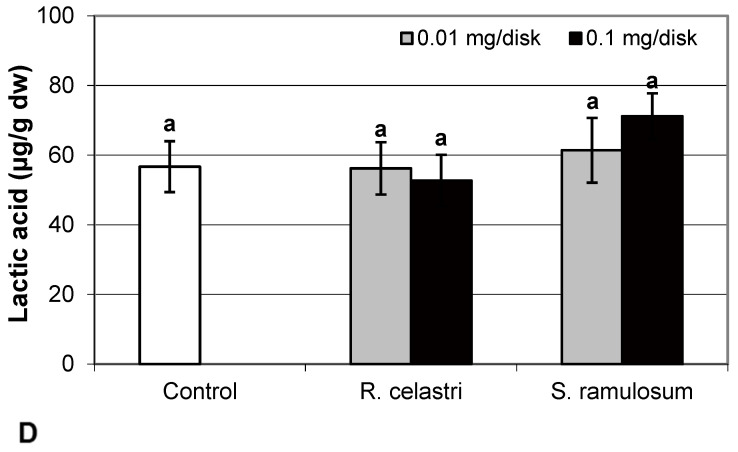
Content of fumaric acid ((**A**); µg/g dw), glutamic acid ((**B**); µg/g dw), ketoglutaric acid ((**C**); µg/g dw), and lactic acid ((**D**); µg/g dw) of 2-week-old photobiont *Asterochloris erici* cultures cultivated on disks with the addition of secondary metabolites extracts from lichens *Ramalina celastri* and *Stereocaulon ramulosum* (0.01 and 0.1 mg/disk). Values in vertical columns followed by the same letter(s) are not significantly different according to Tukey’s test (*p* < 0.05), *n* = 3.

Fumaric acid content (Figure 6A) and lactic acid content (Figure 6D) were stable parameters in photobiont cells and did not change regardless of the lichen extract tested and extract quantities applied. Contents of glutamic acid (Figure 6B) in *A. erici* decreased in a dose-dependent manner for both extracts tested, whereas only the higher doses tested were effective for ketoglutaric acid (Figure 6C).

**Figure 7:**
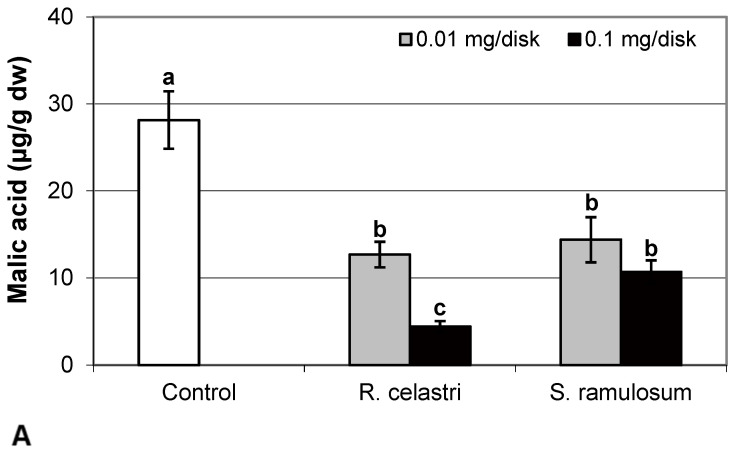

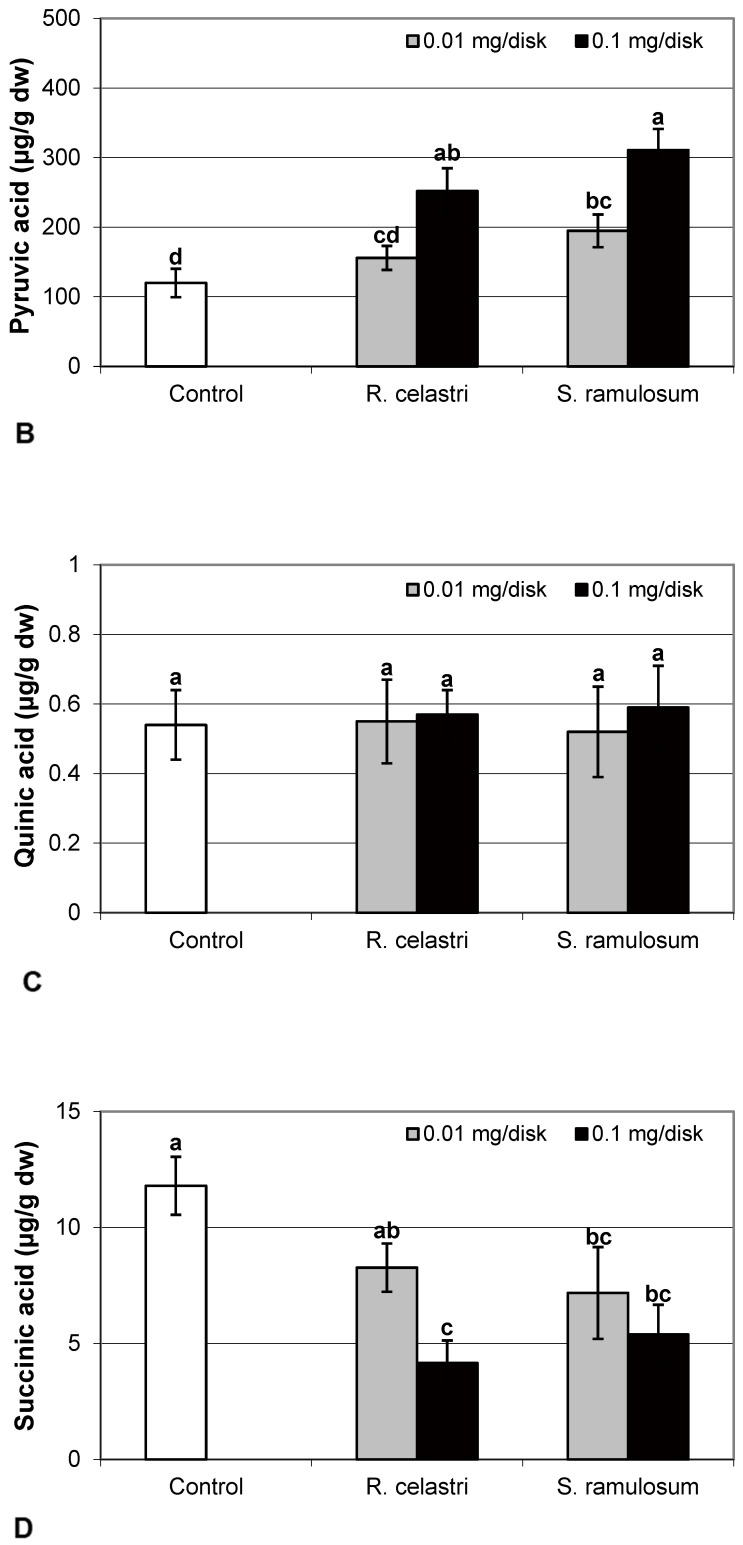
Content of malic acid ((**A**); µg/g dw), pyruvic acid ((**B**); µg/g dw), quinic acid ((**C**); µg/g dw), and succinic acid ((**D**); µg/g dw) of 2-week-old photobiont *Asterochloris erici* cultures cultivated on disks with the addition of secondary metabolites extracts from lichens *Ramalina celastri* and *Stereocaulon ramulosum* (0.01 and 0.1 mg/disk). Values in vertical columns followed by the same letter(s) are not significantly different according to Tukey’s test (*p* < 0.05), *n* = 3.

**Figure 8:**
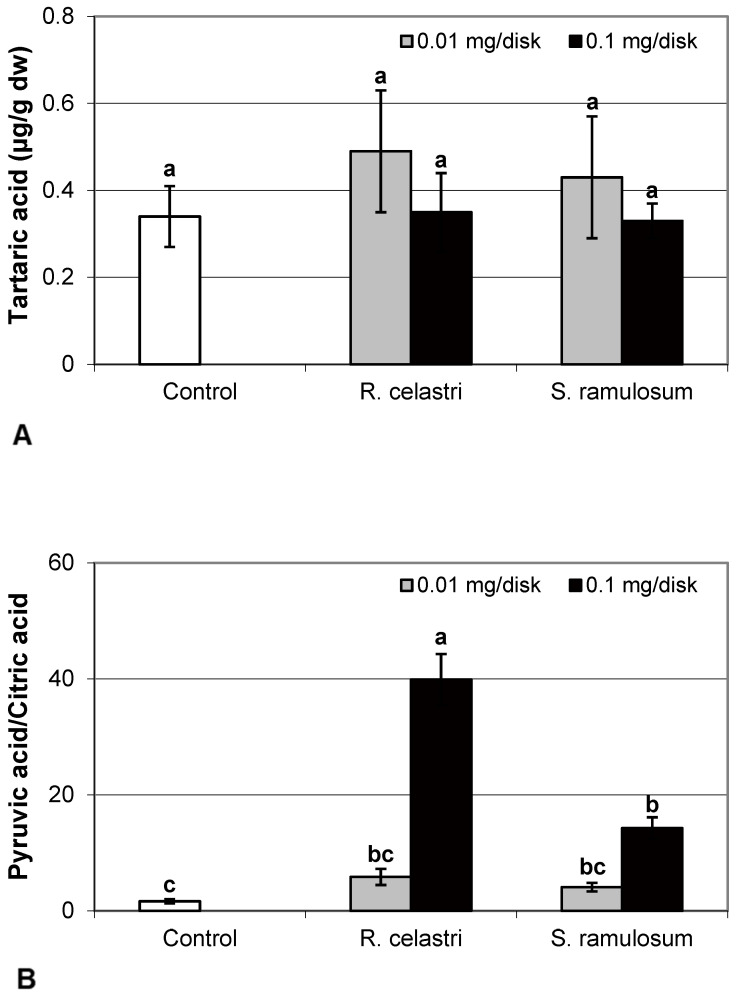
Content of tartaric acid ((**A**); µg/g dw) and pyruvic acid/citric acid (**B**) of 2-week-old photobiont *Asterochloris erici* cultures cultivated on disks with the addition of secondary metabolites extracts from lichens *Ramalina celastri* and *Stereocaulon ramulosum* (0.01 and 0.1 mg/disk). Values in vertical columns followed by the same letter(s) are not significantly different according to Tukey’s test (*p* < 0.05), *n* = 3.

The content of malic acid (Figure 7A) and succinic acid (Figure 7D) in algae decreased as a response to the application of both tested lichen extracts, reflecting the increase in their quantity. For the lower dose of *R. celastri* extracts tested, the decrease in succinic acid was not strong enough to be significant. In contrast, pyruvic acid content in photobiont cells increased with increased tested amounts of extracts (Figure 7B). Contents of quinic acid (Figure 7C) and tartaric acid (Figure 8A) in *A. erici* were stable at all tested amounts of both lichen extracts. The ratio of pyruvic acid/citric acid contents (Figure 8B) in photobionts increased in response to the application of lichen secondary metabolite extracts, more pronounced at extracts from *R. celastri*.

Pyruvic acid ensures the energy to the cells by the citric acid cycle, and it is a key product between catabolism and anabolism of carbohydrates, fats, and proteins. The increase in pyruvate in response to allelochemicals responds similarly to that in response to other xenobiotics (e.g., heavy metals) and is likely directed toward the production of ATP and NADH for other metabolic processes [25]. However, we know almost virtually nothing about the role of organic acids from the Krebs cycle in lichens and their symbionts. 

## 3. Materials and Methods

### 3.1. Organism, Culture Conditions, and Lichen Extracts 

The lichen photobiont *Asterochloris erici* (Ahmadjian) Skaloud et Peksa (syn. *Trebouxia erici* Ahmadjian, UTEX 911), isolated from the lichen *Cladonia cristatella* Tuck., was used in this study. This lichen contains typical secondary metabolites, e.g., usnic acid, barbatic acid, and didimic acid [8]. Photobiont stock cultures were cultivated on agar medium as described in our previous studies [25] and maintained at 22 °C under a 16-h photoperiod and 30 µmol·m^−2^·s^−1^ artificial irradiance (“cool white” tubes). 

For the assessment of the role of lichen secondary metabolites on the growth and metabolism of photobiont cells, acetone extracts from two lichen species were prepared. Usnic acid was isolated from the lichen *Ramalina celastri* (Spreng.) Krog. & Swinscow collected from the stems of Norfolk Island pine trees (*Araucaria heterophylla*), seacoast area near Kiama city, NSW, Australia, on 12 September 2022. The naturally occurring mixture of atranorin and perlatolic acid (approximately 3:1) was obtained from the terrestrial lichen *Stereocaulon ramulosum* (Sw.) Raeusch. also collected at the seacoast near Kiama City, NSW, Australia, on 13 September 2022. Lichens were collected and identified by Prof. Martin Bačkor. Voucher specimens of both lichen species were stored in our laboratory for future reference. The purity of compounds was assessed by High-Performance Liquid Chromatography (HPLC) and Thin Layer Chromatography as described previously [25]. The mean contents of secondary metabolites in lichen *R. celastri* was 0.2% for usnic acid and in *S. ramulosum* was 0.32% for atranorin and 0.11% for perlatolic acid. Identification of secondary metabolites was carried out using the standard of (+)-usnic acid (CAS No.: 7562-61-0, Sigma-Aldrich, St. Louis, MO, USA). Atranorin (CAS No.: 479-20-9) and perlatolic acid (CAS No.: 529-47-5) were isolated according to previous studies performed in our laboratories using a protocol described by Elečko et al. [31].

### 3.2. Allelopathic Assay

Photobionts were cultivated on the surface of glass fiber filter disks (Whatman CF/C filters), 25 mm in diameter, as described in previous studies [25]. 

For quantitative photobiont cultivation, sterilized 25 mm (in diameter) glass fiber disks were subjected to five different pretreatments. Crystals of extracts from lichens *R. celestri* and *S. ramulosum* (0.1 and 0.01 mg/disk) were dissolved in acetone (volume 30 μL) and applied by automatic pipette on the surface of disks while the same volume of acetone was used for control disks. After 4 h of acetone evaporation, individual disks from all treatments were transferred to the surface of solid *Trebouxia* medium in a separate Petri dish, 6 cm in diameter, and 20 µL of algal suspensions were inoculated into the center of each disk. Disk pores allow supplemental nutrient media to pass through the disk and permit growth to be easily determined from changes in biomass. The total mass of cultures was calculated by subtracting the mean fresh weight (fw) of a glass fiber disk saturated by an identical medium from the fw of a disk supporting algal cultures after 14 days of cultivation [25]. Each treatment was replicated twenty times.

### 3.3. Assimilation Pigments and Chlorophyll a Integrity

Weighed disks with grown photobiont cells were directly extracted in the dark for 1 h at 65 °C in 5 mL of dimethylsulfoxide (DMSO). To maximize chlorophyll extraction, cell aggregates were homogenized using mortar; glass fibers of disks facilitated the disruption of the cell walls of algae. After cooling to ambient temperature, the absorbance of the extracts, as a reflection of turbidity, was determined at 750 nm with a spectrophotometer to be certain that it was always less than 0.01. The absorbance of extracts was then read at 665, 649, 435, and 415 nm to assess chlorophyll content and the possibility of chlorophyll *a* degradation [32]. To determine the content of “total” carotenoids, absorbance was read at 480 nm. Chlorophyll *a*, chlorophyll *b*, chlorophyll *a* + *b*, and total carotenoids were calculated using equations derived from specific absorption coefficients for pure chlorophyll *a* and chlorophyll *b* in DMSO. Chlorophyll *a*/*b* was used to assess the physiological competence of algal cells. The ratios of absorbances at 435 and 415 nm, termed the phaeophytinization quotient, were calculated as a reflection of the ratio of chlorophyll *a* to phaeophytin *a* and provide an indication of the integrity of photobiont chlorophyll *a* [33]. Each treatment was replicated three times.

### 3.4. Activity of Photosystem II

Chlorophyll *a* fluorescence was measured in algae grown on glass fiber disks on the surface of *Trebouxia* agar media. While still on the surface of media in Petri dishes to minimize desiccation, photobiont cultures were dark-adapted for 30 min before the measurement. The potential quantum yield of photosystem II (PSII) was measured using a FluorCam MF-800 fluorescence-imaging camera (Photon Systems Instruments Ltd., Brno, Czech Republic) by applying a saturating flash of light 2000 µmol s^−1^ m^−2^ for 1 s. The maximum efficiency of the PSII was assessed by the F_V_/F_M_ ratio. F_V_/F_M_ = (F_M_ − F_0_)/F_M_, where F_0_ represents ground fluorescence in the dark-adapted state and F_M_ represents maximum fluorescence at a saturating radiation pulse in the dark-adapted state. Chlorophyll fluorescence parameters were taken from three separate positions on each disk, and the mean value was used as the overall observation [25]. Each treatment was replicated three times.

### 3.5. Content of Soluble Proteins

Photobiont cultures grown on Whatman CF/C disks were homogenized directly with the disks in an ice-cold mortar in phosphate buffer (50 mM). After centrifugation at 10,000× *g* at 4 °C for 20 min, the water-soluble protein content of supernatants was measured using the methods of Bradford [34]. Supernatants (100 μL) were pipetted into 900 μL of Bradford assay kit (Bio-Rad, Hercules, CA, USA) in a spectrophotometric cuvette and mixed. After 10 min, the absorbance of samples was spectrophotometrically measured at 595 nm. Bovine serum albumin was used as a calibration standard [33]. Each treatment was replicated three times.

### 3.6. Oxidative Status

Hydrogen peroxide and superoxide were measured in homogenates with potassium phosphate buffer prepared for assay of determination of soluble proteins. Hydrogen peroxide was quantified by the TiCl_4_ method, and superoxide was estimated by monitoring the formation of nitrite from hydroxylamine at 530 nm [16]. The reaction mixture contained 0.27 mL of potassium phosphate buffer, 0.03 mL of 10 mM hydroxylamine, 0.3 mL of supernatant, 0.3 mL of 17 mM sulfanilamide, 0.3 mL of 7 mM α-naphthylamine, and 0.3 mL of diethyl ether. Each treatment was replicated three times.

### 3.7. Membrane Lipid Peroxidation 

The membrane lipid peroxidation state in photobiont cultures was estimated using the thiobarbituric acid reactive substances assay (TBARS) as described in [33]. Algae grown on Whatman CF/C disks were homogenized with disks in a mortar using ice-cold 10% (*w*/*v*) trichloroacetic acid (TCA). The homogenate (2 mL final volume) was centrifuged at 10,000× *g* for 10 min. The supernatant (1 mL) was added to 1 mL of 0.6% thiobarbituric acid (TBA) in 10% TCA. After the treatment of samples at 98 °C for 25 min and immediate cooling in an ice bath, the mixture was again centrifuged at 10,000× *g* for 10 min. The absorbance of the supernatant was measured at 532 nm (extinction coefficient for MDA-TBA complex 155 mM^−1^ cm^−1^) and corrected for non-specific absorption at 600 nm. Three replicates were used for each variant of the experiment.

### 3.8. Determination of Ascorbic Acid (AsA), Glutathione (GSH, GSSG) and Organic Acids

Reduced (GSH), oxidized glutathione (GSSG), and AsA were extracted from photobiont samples with 0.1 M HCl (0.1 g fw/1 mL) and quantified using LC–MS/MS (Agilent 1200 Series Rapid Resolution LC system coupled on-line to an MS detector Agilent 6460 Triple Quadrupole with Agilent Jet Stream Technologies, Santa Clara, CA, USA) at *m*/*z* values 308/76, 613/231, and 177/95 in positive MRM mode, respectively. Separation was conducted using column Zorbax EC-C18 100 × 4.6 mm, 2.7 µm particle size, and a mobile phase consisting of 0.2% acetic acid and methanol (95:5). The flow rate was 0.6 mL/min, and the column temperature was set at 25 °C. Freshly prepared standards were used for calibration and quantification [35].

Liquid chromatography with tandem mass spectrometry using a triple-quadrupole MS detector was used to analyze organic acids in the algae. A volume of 1 mL of 80% methanol and glass beads 0.5 mm in diameter were added to samples of algal cells. Samples were homogenized at 6800× *g*, centrifuged at 16,000× *g*, and then filtered through Whatman Mini-UniPrep syringeless filters at 0.45 μm before analysis by liquid chromatography with mass detection. The samples were analyzed using an Agilent 1200 Rapid Resolution LC system. The system was connected to an Agilent Technologies 6460 triple-quadrupole MS detector with an Agilent Jet Stream, all from Agilent Technologies, Waldbronn, Germany. This system is described in more detail in previous publications [17,25,35].

### 3.9. Statistical Analysis

One-way analysis of variance and Tukey’s pairwise comparisons (MINITAB Release 17, 2017, Minitab Inc., State College, PA, USA) were used to determine the significance (*p* < 0.05) of differences in all measured parameters.

## 4. Conclusions

As we noted previously, lichens are self-sustaining ecosystems forming thallus, which is composed of a fungal partner and an extracellular arrangement of minimally one photosynthetic partner, alga, or/and cyanobacteria. There may also be an indeterminate number of other microscopic organisms within the thallus that are of greater or lesser importance to the functioning of the thallus as a “single” unit. Lichen photobionts are in permanent contact with their mycobionts, and lichen symbiosis is a long-term association (some lichens may survive for hundreds of years); we assume that the production of typical secondary metabolites by mycobionts is a key element for maintaining homeostasis in lichen thallus, preventing overgrowth of biomass of photobionts resulting in an imbalance between bionts. 

Although the biological and ecological roles of lichen secondary metabolites are diverse, due to a high number of so far discovered chemical compounds and their origin, some of them possess allelopathic effects. The phytotoxicity of lichen compounds to algal partners of symbiosis has only been investigated in a few studies so far. Toxicity symptoms involve growth (biomass production), inhibition of photobionts, alteration of the composition of assimilation pigments, a decrease in chlorophyll *a* fluorescence, and chlorophyll *a* integrity. Phytotoxicity of lichen secondary metabolites is manifested with increased production of ROS and peroxidation of membrane lipids. Regulation of the antioxidant defense system in the *A. erici* photobiont is followed by decreased content of ascorbic acid in the cells and decreased contents of reduced and oxidized glutathione. Contents of selected organic acids involved in the Krebs cycle were altered in photobiont cells exposed to crystals of lichen substances. One of the most prominent markers of phytotoxicity of lichen substances in lichen algae was the increased pyruvic acid/citric acid ratio employed in the present study. 

## Data Availability

The data will be provided upon request.

## Abbreviations

AsA—ascorbic acid; ATR—atranorin; dw—dry weight; fw—fresh weight; GSH—reduced glutathione; GSSG—oxidized glutathione; PA—perlotolic acid; RNS—reactive nitrogen species; ROS—reactive oxygen species; TBARS—thiobarbituric acid reactive substances; UA—usnic acid.
